# Nutritional Status and Body Composition in Patients Suffering From Chronic Respiratory Diseases and Its Correlation With Pulmonary Rehabilitation

**DOI:** 10.3389/fresc.2021.725534

**Published:** 2021-12-06

**Authors:** Emiel F. M. Wouters

**Affiliations:** ^1^Ludwig Boltzmann Institute for Lung Health, Vienna, Austria; ^2^Department of Respiratory Medicine, Maastricht University Medical Center, Maastricht, Netherlands

**Keywords:** nutrition, body composition, COPD, chronic respiratory conditions, rehabilitation, muscle function

## Abstract

As part of an individualized intervention to improve the physical, emotional, and social functioning of patients with chronic respiratory diseases in general and chronic obstructive pulmonary disease in particular, awareness of the presence and consequences of changes in body composition increased enormously during the last decades, and nutritional intervention is considered as an essential component in the comprehensive approach of these patients. This review describes the prevalence and the clinical impact of body composition changes and also provides an update of current intervention strategies. It is argued that body composition, preferentially a three-component evaluation of fat, lean, and bone mass, must become part of a thorough assessment of every patient, admitted for pulmonary rehabilitation.

## Introduction

In the first authoritative statement on pulmonary rehabilitation in 1974, this intervention was introduced as an art of medical practice wherein an individually tailored, multidisciplinary program is formulated which, through adequate diagnosis, therapy, emotional support, and education, stabilized or reversed both the physio and psychopathology of pulmonary diseases and attempted to return the patient to the highest possible capacity allowed by his pulmonary handicap and overall life situation (1). In the absence of relevant reversibility of the underlying respiratory pathology in patients suffering from chronic respiratory diseases, skepticism grew toward the rationale and outcomes of pulmonary rehabilitation in these patients; exercise tolerance was considered to be limited by the lung impairment, and exercise conditioning did not improve lung function (2). Furthermore, it was doubted that patients with chronic respiratory impairment could exercise to a sufficient intensity to exceed a critical training threshold to improve muscle function. Different landmark studies demonstrated that rigorous exercise training in these patients resulted in substantial improvements in exercise tolerance and physiological training effects (3, 4). These studies contributed to a shift to the role of skeletal muscles even in patients with chronic respiratory conditions.

Although all definitions of pulmonary rehabilitation are targeted to patients with chronic respiratory conditions, at present, patients with chronic obstructive pulmonary disease (COPD) form the most important target population of these programs. Back in 2001, COPD was defined as a disease state characterized by airflow limitation that is not fully reversible (5). At present, driven by systematic diagnostic work-up as part of pulmonary rehabilitation programs, COPD is now recognized as a complex condition with many different components and mechanisms contributing to its pathophysiology and clinical burden, and the role of comorbidities on the burden and mortality is now well recognized (6).

In the latest American Thoracic Society/European Respiratory Society statement, body composition abnormalities and interventions are considered a key component of a patient-tailored comprehensive approach of patients with chronic respiratory impairment to contribute to an improvement in the physical condition, in particular (7). A survey on organizational aspects of pulmonary rehabilitation programs in Europe and North America demonstrated that nutritional support, supervised by a dietician, was part of the rehabilitation program in 76.1 and 93.6% of the programs (8).

This review describes the prevalence and the assessment and clinical and functional impact of body weight and body composition changes in COPD patients, in particular. Nutritional support strategies and the outcomes of nutraceuticals as an ergogenic aid in these patients are reviewed.

## Assessment of Body Composition

Unintended weight loss is broadly accepted as an indicator of an inevitable and fatal progression in different disease processes, including COPD. As a rule of thumb, involuntary weight loss >5% during the last 6 months is considered clinically significant. Abnormalities in weight are traditionally classified on the basis of the body mass index (BMI, body weight in kilograms divided by height in meters squared) as underweight (<21 kg/m^2^), normal weight (21–25 kg/m^2^), overweight (> 25–30 kg/m^2^) and obese (>30 kg/m^2^). Recent large population studies revealed that the age-standardized rate of death from any cause is lowest among participants with a BMI of 22.5–24.9 kg/m^2^ and of 20–25 kg/m^2^ in analyses restricted to never-smokers (9, 10). The hazard ratio per 5 kg/m^2^ units higher BMI decreases at older age (11). For most diseases, including chronic respiratory conditions, a J-shaped association of overall mortality is reported with BMI, with the lowest risk occurring in the range 21–25 kg/m^2^: BMI associated with lowest mortality risk is higher in older individuals than in younger individuals (12). In patients with moderate to severe airflow limitation, a BMI <25 kg/m^2^ is consistently associated with increased mortality risk relative to overweight and obese patients (13–15). A dose-response meta-analysis involving 30,182 patients with COPD confirmed that overweight is associated with a lower risk of all-cause mortality whereas underweight is associated with higher risk (16). After the publication of the so-called bodyweight, airflow obstruction, dyspnea, and exercise capacity (BODE) index, the impact of multidimensional assessment has been widely accepted (17).

Weight changes and BMI classification do not take into account body compositional shifts, including fat and fat-free mass. In clinical practice, there is no standard method for the assessment of body composition. Many reports are based on a two-compartment model distinguishing between fat (FM) and fat-free mass (FFM). FFM is further divided into an intracellular compartment, reflecting muscle mass and other metabolizing tissues, and an extracellular compartment. FFM is commonly used as an indirect measure of muscle mass. Different descriptors of low muscle mass are used interchangeably in the literature. Bio-electrical impedance analysis is widely used as a valid and reproducible method to assess FM and FFM, particularly in patients with COPD (18). In normal to underweight patients with COPD, the 10th percentile of age- and sex-adjusted FFM index (FFMI: FFM/height^2^) is defined as abnormally low. In Caucasian patients, this corresponds to an FFMI of 17 kg/m^2^ for men and <15 kg/m^2^ for women as clinically useful proxies (19). These cut-offs are probably too low to detect an abnormally low FFMI in overweight and obese populations. Indeed, low FFMI could not be identified when these proxies are applied (20). New BMI-specific reference values are derived in particular for overweight and obese populations (21).

Particularly in patients with COPD admitted for pulmonary rehabilitation, the prevalence of nutritional depletion is very high (45%) and loss of FFM could be masked by a normal BMI (22). In a large out-patient population of patients with COPD, the prevalence of low FFMI was present in 27% of the population; low FFMI was masked by a normal BMI of 15% (23). Most data are reported from cross-sectional studies. In the evaluation of COPD Longitudinally to Identify Predictive Surrogate End (ECLIPSE) study, comparing patients with COPD with smoking and non-smoking controls, low muscle mass was present in 20% of patients with COPD and 9 and 4% of the smoking and non-smoking control subjects, respectively. Interestingly, changes in body composition over the 3-year follow-up period were small and comparable among the three groups (24).

A more precise analysis of body composition can be performed by describing a three-compartment model consisting of the tissue compartments fat mass, lean mass (LM), and bone mineral content (BMC). FFM is composed of BMC and LM. Dual X-ray absorptiometry (DXA) is a validated tool to investigate body composition phenotypes as it precisely analyses the amount of BMC and soft tissue (FM and LM) of the whole body and in specific anatomical regions (25, 26). Particularly, LM measured at the limbs is a marker of skeletal muscle mass and therefore important in the assessment of muscle depletion (27). Sarcopenia is defined as low skeletal muscle mass based on the assessment of the lean appendicular mass (28–31). Appendicular skeletal muscle mass, assessed by DXA, seems a better indicator for muscle and functional dysfunction than overall FFM (32).

Reference values of body composition by DXA in adults aged 18–81 years are recently reported (33). A recent systematic review and meta-analysis reported the prevalence of sarcopenia, based on low muscle mass and decreased muscle function in 21.6% of patients with COPD, ranging from 8% in population-based to 21% in clinic-based studies and 63% in patients with COPD residing in nursing homes (34).

### Clinical and Functional Impact of Body Weight and Body Composition in COPD

Reduction in symptoms, improvement in exercise performance, and also in health status can be defined as outcomes for COPD management in general and pulmonary rehabilitation in particular. A thorough understanding of the impact of body weight and body composition on these traits can guide interventions to improve functional and health status in these patients.

Exploring the association of functional measures and BMI as a measure of body composition is limited because BMI does not distinguish between FFM and FM. Reported data on the relation between exercise performance and BMI are therefore difficult to interpret. In the 1980s, positive associations were reported between nutritional status, measured by body weight and expressed as a percentage of ideal weight, and maximal exercise performance (35, 36). Others could not find differences in field exercise tests, such as the 6-min walking test between underweight and normal weight patients with COPD (35, 37), whereas other studies reported significant positive associations between these field exercise tests and body weight (38). However, those associations became significantly better between fat-free mass and walking distance (38). Later on, these data were confirmed in clinically stable COPD out-patients (39).

A detailed study of exercise tolerance in undernourished patients with COPD, based on the percentage ideal body weight, is reported by Palange et al. in 1995 (40). They found that undernourished patients with COPD showed a greater reduction in maximal workload and peak O_2_ uptake with an earlier onset of metabolic acidosis. In addition, indices reflecting the O_2_ cost of ventilation were higher in undernourished patients with COPD. Nutritional status correlated with the exercise-tolerance onset of metabolic acidosis and with the dead space/tidal volume ratio. The authors concluded that undernutrition affects muscle aerobic capacity and exercise tolerance, and was associated with high wasted ventilation and oxygen cost of ventilation (40). Later on, in weight-stable patients with COPD admitted for pulmonary rehabilitation, Franssen et al. reported that FFM explained 38% of the variation in VO_2_ max and 56% in combination with age and DLco. FFM, age, FEV_1_, and DLco all individually contributed to achievable maximal load (41).

Loss of muscle mass will influence the strength of the skeletal muscles, reduction in muscle bulk results in a decrease in muscle force output, whereas the mechanical effectiveness of the residual myofibrillar material remains unaffected. Intriguingly, in undernourished patients with the gastro-intestinal disease, Lopes and colleagues reported already in 1982 an increased muscle fatiguability and slowing of the relaxation of the adductor pollicis muscle, possibly related to a decrease in local energy stores of the limb muscles (42). Others reported a reduced strength of the sternomastoid muscle and an increase in oxygen consumption of the ventilatory muscles in underweight patients with COPD (37, 43). Muscle function corrected for total FFM was well-preserved in patients with COPD and did not differ from controls, fitting with preserved contractility (44).

Considering the impact of ventilatory pump dysfunction on the experience of dyspnea in patients with COPD, respiratory muscle strength was considered as an important measure and outcome. Respiratory muscle strength was found to correlate significantly with body weight and FFM (38, 45). Others focused attention on the impact of skeletal muscle weakness on exercise limitation in patients with COPD (46, 47). Franssen et al. extended these reduced skeletal muscle functions to the upper limbs, and they showed that quadriceps muscle strength was more impaired in FFM-depleted patients with COPD, with the preservation of biceps strength. Furthermore, they demonstrated that loss of muscle endurance was restricted to the lower limbs independent of FFM, where muscle endurance reflects the capacity of the muscle to sustain mechanical output (48).

In a large cohort of stable patients with COPD, the impact of FFM on upper arm extremity performance was supported by demonstrating a significant relationship between FFM and hand-grip strength (23). Others confirmed that body composition assessment gave valuable information about hand grip and respiratory muscle functioning (49). A new method to approach body composition is to use measures of muscle and fat structures captured as part of body imaging as surrogates of body composition (50). It was demonstrated that hand-grip strength correlated significantly with the pectoralis muscle area and with subcutaneous adipose tissue, but not with body mass index (51). Intriguingly, the same study demonstrated that hand-grip strength was associated with exacerbation risk (51). Assessment of muscle weakness can guide prognosis in these patients as different studies reported an association between muscle endurance and strength and mortality risk (52, 53).

Despite the fact that health status has been used extensively in descriptive and therapeutic evaluation studies in patients with COPD in general, and as an outcome of pulmonary rehabilitation in particular, the factors determining the score of health status are studied and understood poorly. Already 20 years ago, cross-sectional reports focused their attention on the impact of low muscle mass on symptoms, activity, and impact as domains of health status (54, 55). Others focused on the relationship between health status and muscle strength and endurance in patients with COPD (56). Montes de Oca et al. reported in 2006 that impaired health status is related to peripheral muscle changes, characterized by less type 1 fiber proportion (57). Intriguingly are the data reported by Huber et al. demonstrating the non-linear relationship between BMI and health status in patients with COPD. They reported more impaired health status in the overweight and obese Global strategy for the Diagnosis, Management, and Prevention of Chronic Obstructive Pulmonary Disease (GOLD) one to three patients, whereas obese GOLD four patients with COPD reported a better health status than their normal-weight peers.

Body composition assessment may also be a predictor of the type of respiratory impairment as well as of the multimorbidity pattern in patients with COPD. Substantial differences in body composition between emphysematous and non-emphysematous COPD patients were already reported in 1999 (58). Recent findings based on the ECLIPSE data set confirmed that patients with more emphysema undergo excessive loss of pulmonary as well as extra-pulmonary tissue, and even the multiorgan loss of tissue COPD phenotype was suggested to identify these subgroups of patients with COPD (59). By clustering the multimorbid involvement in patients with COPD, a so-called cachectic cluster could be identified, based on a higher prevalence of underweight, low muscle mass, osteoporosis, and renal impairment (60). Others reported that this cluster seems specific for a COPD population, compared with an age-matched control group (61).

Body composition also predicts prognosis and mortality in COPD patients. Indeed, survival studies in selected groups of patients with COPD have shown higher mortality in underweight and normal weight patients with COPD (13, 15, 62). A large-scale prospective community-based cohort study in Japanese men confirmed that lower BMI and greater weight loss are associated with a greater risk of mortality (63). A dose-response meta-analysis of BMI and mortality in COPD patients indicated that overweight is associated with a lower risk of all-cause mortality, whereas underweight is associated with a higher risk of all-cause mortality (16). Particularly, fat-free mass is an independent risk factor of mortality in these patients (64). In this context, data on the relationship between mid-thigh cross-sectional area and increased mortality remain intriguing (65).

Considering that the goal of pulmonary rehabilitation is to offer an individualized intervention based on a thorough assessment, the reported data illustrate the importance of a two-compartment body composition assessment to understand the clinical burden experienced by the patient and to adequately plan intervention strategies. A three-compartment body composition assessment would be advisable to evaluate appendicular muscle mass as well as bone mass in these patients. Finally, simple muscle function testing, such as hand-grip strength, will be a good surrogate marker for muscle bulk and could guide interventions to improve or maintain functional status in these patients. Combined assessment of muscle mass and function will contribute to the early detection of sarcopenia in these patients.

## Nutritional Support

Although the importance of nutrition in health and disease is intuitively acknowledged, the role of nutrition in the management of chronic respiratory conditions in general, and COPD in particular, has only gained interest during the last decades. Traditionally, weight loss was considered to be an inevitable and irreversible terminal event related to the severity of the disease process, and weight loss was even considered as an adaptive mechanism to decrease oxygen consumption.

Weight loss occurs when energy balance is negative and occurs when energy expenditure exceeds dietary intake. Total daily energy expenditure (TDE) is usually divided into three components: (1) resting energy expenditure (REE); comprising sleeping metabolic rate and the energy cost of arousal; (2) diet-induced thermogenesis; and (3) physical activity-induced thermogenesis. REE is found to be elevated in 25% of patients with COPD. Drug therapy, especially the use of beta-2-agonists, increased work of breathing, and also the presence of systemic inflammation have been related to this increase in REE (66–69). Diet-induced thermogenesis represents metabolic oxygen cost for the processing of ingested nutrients. This thermic effect of dietary intake remains unclear (70, 71). Studies demonstrate that patients with COPD have a significantly higher TDE than normal, particularly related to the higher activity-related component (72). Indeed, no differences in TDE are reported between hypermetabolic and normal metabolic patients (73). Mechanical inefficiency of leg exercise, increased ventilatory demand related to the work of breathing, or inefficient muscle metabolism can contribute to this increased TDE (72, 74, 75). Indirect evidence for altered energy expenditure in patients with COPD is a rise in plasma ammonia even during low-intensity walking, an indicator of muscle ATP depletion and metabolic stress (76).

The first clinical trials investigating the efficacy of short-term (2–3 weeks) nutritional intervention consisted of nutritional supplementation by means of oral liquid supplements or enteral nutrition. These short-term studies showed that nutritional supplementation leads to a significant increase in body weight and respiratory muscle function (77, 78). Significant improvements in respiratory and peripheral skeletal muscle function, exercise capacity, and health status were also observed in one in-patient and one out-patient study following a 3-months oral supplementation of about 1,000 kcal daily (37, 79). The problem of most clinical trials of nutrition supplementation is that the sample size is rather small, characterization of patients is limited, and shifts in energy intake between supplements and regular dietary intake are poorly documented. Ferreira et al., therefore, conducted a Cochrane review of 17 trials of >2 weeks of nutritional support. The authors conclude that nutritional supplementation promotes weight gain among patients with COPD. In addition, significant improvements are reported in anthropometric measures, 6-min walking distance, respiratory muscle function, and overall health status in undernourished patients with COPD (80). Recent meta-analyses broadly confirmed these findings; positive results are also reported for total energy intake, handgrip, and quadriceps strength (81, 82).

In case of non-response, biological characteristics underlying the disease process of COPD must be considered. Aging, relative anorexia, and an elevated systemic inflammatory response have been identified as determining factors (83).

Energy- and protein-enriched diets are now generally recommended; protein should provide 20% of the total energy intake (84). Suitable energy- and protein-enriched diet can be achieved by several small portions spread throughout the day; a dietician can tailor the diet taking into account eating habits, lifestyle, symptoms, likes and dislikes of the subject (85).

To create an anabolic stimulus to increase protein mass, the combination of nutritional intervention with even a comprehensive rehabilitation program was advocated (86). Indeed, most studies with FFM as the outcome, have combined nutritional supplementation with exercise (80). Previous studies combining pulmonary rehabilitation with a hormonal anabolic intervention have demonstrated a higher increase in FFM and intracellular mass (87). In normal-weight patients with COPD, a small increase in FFM (1 kg FFM difference) is also reported after pulmonary rehabilitation (41). Therefore, it can be questioned whether exercise can induce the reported effects on FFM, independent of nutritional intervention. Considering the multimodality of cachexia in COPD patients, targeted-medical nutrition (TMN) studies are conducted. A randomized controlled study compared TMN containing high-dose omega 3-fatty acids, vitamin D, and high-quality protein with an isocaloric comparator in patients with COPD with involuntary weight loss or low BMI. TMN was well tolerated with a good safety profile and positive effects on blood pressure and blood lipids and exercise-induced fatigue and dyspnea (88). Another study reported that oral supplementation enriched with leucine, vitamin D, and omega-3 fatty acids has positive effects on nutritional status, inspiratory muscle strength, and physical activity compared with placebo (89).

The clinical relevance of positive treatment response is supported by a *post-hoc* survival analysis demonstrating that a weight gain > 2 kg in depleted and non-depleted FFM patients and an increase in respiratory muscle strength were associated with significantly increased survival rates.

The cost-effectiveness of nutritional supplementation is poorly studied in patients with COPD although numerous studies reported that being undernourished is associated with longer in-patient hospital stays, a higher probability of being readmitted, and an increase in healthcare utilisation (90–93). Two studies could not demonstrate differences in health care utilization (94, 95). The 24-month interdisciplinary community-based COPD management program trial comparing nutritional rehabilitation with usual care in patients with COPD with low muscle mass reported a significant reduction in hospital costs for the intervention group (96). Therefore, it can be concluded that the negative effect of low body weight can be reversed by appropriate nutritional intervention in patients with COPD (13).

### Nutraceuticals as an Ergogenic Aid

In analogy with the role of nutrition in the fields of sports and medicine, the scope of nutritional intervention moved to enhance physical performance in patients with COPD. A lot of small-scale, single-center intervention studies are published in the literature. Dairy proteins, and in particular casein, resulted in protein anabolism during and following exercise in patients with COPD (97). A pilot study found that pressurized whey in combination with exercise training may potentiate the effects of exercise training alone on exercise tolerance and quality of life (98). In cancer patients and patients with cystic fibrosis, dietary free essential amino acids seem very efficient in inducing anabolism independent of the presence of muscle or recent weight loss (99, 100). In patients with COPD, a free essential amino acid mixture with a high proportion (40%) of leucine stimulates whole-body protein metabolism more than free amino acid supplements with the composition of complete proteins; effects did not differ between patients with COPD and healthy older subjects (101).

Independent of the degree of FFM depletion, intrinsic abnormalities in peripheral skeletal muscle morphology and metabolism are described pointing toward a decreased oxidative capacity. A well-established qualitative alteration is the loss of oxidative phenotype characterized by a muscle fiber type I to type II shift and a loss of oxidative capacity (102). Besides these fiber type shifts, decreased levels of muscle oxidative metabolic markers and nutrient sensing regulators of cellular energy state are reported (103). Interventions with poly-unsaturated fatty acids (PUFA) can upregulate fat oxidative gene expression by the activation of peroxisome proliferators activated receptors (PPARs) (104). PPARs promote the uptake of circulating fatty acids by cells through upregulation of the lipoprotein lipase gene (105). In addition, PPARs control the mitochondrial fatty acid import and beta-oxidation (106). Broekhuizen et al. reported an increased peak load in the incremental exercise test as well as an increased duration of the constant work rate test after PUFA intervention (107). Others reported that oral nutritional supplementation, enriched with leucine and PUFA, has additional effects on nutritional status, inspiratory muscle function, and physical activity (89). However, a recent systematic review concluded that there is still insufficient evidence to confirm a relationship between long-chain PUFA intake and the prevalence, severity, or outcomes in COPD (108). Moreover, a recent Chinese study reports that the concentrations of n-3 and n-6 polyunsaturated fatty acids increase over time along with the progression of COPD (109).

Creatine is a widely available nutritional supplement. When phosphorylated, creatine forms a substrate for the generation of adenosine triphosphate, the basic unit for energy generation. Oral supplements with creatine have been used to enhance gains in muscle function and mass. Small randomized controlled trials have been performed in patients with COPD receiving pulmonary rehabilitation; results are conflicting (110–112). A systematic review and meta-analysis of reported data show that creatine supplementation during pulmonary rehabilitation in patients with COPD does not improve exercise capacity, health status, or muscle strength. Based on these findings, creatine supplementation cannot be recommended as an adjunct for pulmonary rehabilitation (113).

Dietary nitrate has been shown to be a nutraceutical that can improve exercise capacity in young healthy individuals (114). Oxygen cost of breathing has been shown to decrease after dietary nitrate intake, without affecting resting metabolic rate (115). Dietary nitrate is reduced in the body to nitrite, which subsequently is converted to nitric oxide (NO) (116). Beetroot juice contains high quantities of nitrate (117). NO availability may modulate blood pressure as well as muscle-related processes including muscle contractility, glucose homeostasis, blood flow, mitochondrial respiration, and biogenesis (118). NO improves the mitochondrial efficiency of energy production per unit of O_2_ by limiting proton leakage in the respiratory chain (119). The nitrate–NO pathway forms also a backup system for NO production, particularly during hypoxic conditions (116). Given that patients with COPD experience greater degrees of tissue hypoxia and have a decreased exercise capacity, it was hypothesized that dietary nitrate supplementation would improve mechanical efficiency and exercise performance. Outcomes of different small-scale studies are conflicting; some studies report an increase in median exercise time during constant submaximal exercise testing while others could not confirm an enhanced endurance exercise performance, despite a reduced oxygen consumption at isotime (120, 121). Others reported an increase in the incremental shuttle walk distance after high nitrate intake, (122) whereas in other studies any difference in exercise endurance, functional walking capacity, O_2_ consumption during submaximal cycling, or physical activity level in patients with COPD could be demonstrated (123, 124). Beijers et al. confirmed that acute and a 7-day sodium nitrate supplementation does not modulate mechanical efficiency in COPD patients (125).

In conclusion, despite the appealing biochemical and biological action of these nutraceuticals to improve exercise performance and functional status, published small-scale studies offer no evidence to introduce these interventions in clinical practice for patients with chronic respiratory conditions.

### Pathobiology of Anabolic and Catabolic Pathways in COPD

A variety of triggers can induce loss of muscle mass: hypoxemia, oxidative stress, inflammation, impaired growth signaling, oral glucocorticoids, disuse, and malnutrition as well as smoking (126, 127). The interplay of these triggers during disease journeys in general and COPD, in particular, is poorly documented as most information is derived from cross-sectional muscle biopsies, most frequently from the quadriceps muscle. Numerous studies reported new insights in the molecular regulation of anabolic and catabolic pathways in the skeletal muscle (128). Studies demonstrate an increased catabolic signaling by NF-κB and fork-head box O transcription factor (FOXO) in COPD in general and even increased in cachectic COPD patients (129–131). This increased catabolic signaling through FOXO and NF-κB can induce gene expression of key factors in both the ubiquitin-proteasome system (UPS) and the autophagy-lysosome pathway (127, 132). The ubiquitin 26S-proteasome pathway consists of coordinated actions of the ubiquitin-conjugating and ligating enzymes that link ubiquitin chains onto proteins to mark them for degradation by the proteasome (133). Different studies report an increase in UPS activity in limb muscles. This UPS activity seems enhanced in cachectic COPD patients (128, 134–136). Autophagy is another highly conserved degradation process in which portions of cytosol and organelles are sequestered into a double-membrane vesicle, an autophagosome, and delivered into a degradative organelle, the vacuole/lysosome, for breakdown and recycling of the resulting macromolecules (137). Its involvement in COPD and loss of muscle mass in particular needs further investigation (128). Considering the muscular protein synthesis signaling, the overall findings are an increase in protein synthesis signaling in limb muscles and even more in respiratory muscles in patients with COPD and even more in the cachectic patients (128, 131, 134, 135, 138). Besides the turnover of proteins, the turnover of myonuclei appears essential for muscle regeneration (139). The role of apoptosis in skeletal muscles of COPD patients remains inconclusive; increased apoptosis in the limb and diaphragm of cachectic COPD patients is reported (136, 140) whereas others could not find differences in the case of maintained muscle mass (141). No differences are yet reported in the number of satellite cells (142). No differences in myostatin, a negative regulator of myogenesis, are reported in cachectic patients with COPD (129, 131, 143).

In conclusion, although very challenging to combat muscle wasting by targeting key pathways as precision medicine intervention, current therapeutic perspectives are largely unclear.

## Summary and a General Conclusion

Body composition has to become an essential component of a thorough assessment of patients admitted for pulmonary rehabilitation. A three-compartment body composition assessment not only allows to measure fat and fat-free mass but also to compartmentalize into subcutaneous and visceral fat, to identify appendicular loss of skeletal muscle mass and even changes in bone mass. Body composition measurement can be supported by simple muscle function tests as a handgrip and/or quadriceps strength. At present, nutritional intervention combined with exercise training must be offered to patients with low body weight and involuntary weight loss. Future intervention studies must apply a clear taxonomy of body composition changes and must standardize functional outcome measures.

## Author Contributions

The author confirms being the sole contributor of this work and has approved it for publication.

## Conflict of Interest

The author declares that the research was conducted in the absence of any commercial or financial relationships that could be construed as a potential conflict of interest.

## Publisher's Note

All claims expressed in this article are solely those of the authors and do not necessarily represent those of their affiliated organizations, or those of the publisher, the editors and the reviewers. Any product that may be evaluated in this article, or claim that may be made by its manufacturer, is not guaranteed or endorsed by the publisher.
